# Rehabilitation combined with neural progenitor cell grafts enables functional recovery in chronic spinal cord injury

**DOI:** 10.1172/jci.insight.158000

**Published:** 2022-08-22

**Authors:** Paul Lu, Camila M. Freria, Lori Graham, Amanda N. Tran, Ashley Villarta, Dena Yassin, J. Russell Huie, Adam R. Ferguson, Mark H. Tuszynski

**Affiliations:** 1 Veterans Administration Medical Center, San Diego, California, USA.; 2 Department of Neurosciences, University of California, San Diego, La Jolla, California, USA.; 3 Department of Neurological Surgery, University of California, San Francisco, California, USA.

**Keywords:** Neuroscience, Stem cell transplantation

## Abstract

We reported previously that neural progenitor cell (NPC) grafts form neural relays across sites of subacute spinal cord injury (SCI) and support functional recovery. Here, we examine whether NPC grafts after *chronic* delays also support recovery and whether intensive rehabilitation further enhances recovery. One month after severe bilateral cervical contusion, rats received daily intensive rehabilitation, NPC grafts, or both rehabilitation and grafts. Notably, only the combination of rehabilitation and grafting significantly improved functional recovery. Moreover, improved functional outcomes were associated with a rehabilitation-induced increase in host corticospinal axon regeneration into grafts. These findings identify a critical and synergistic role of rehabilitation and neural stem cell therapy in driving neural plasticity to support functional recovery after chronic and severe SCI.

## Introduction

Spinal cord injury (SCI) afflicts more than 2 million people worldwide (1) and lacks effective proregenerative therapies. Recently, neural stem cell (NSC) and neural progenitor cell (NPC) grafts have been reported to support host axon regeneration and synapse formation with grafted neurons in the lesion site, while axons from grafted neurons extend axons and form synapses with host circuits distal to the lesion (2–5). In both rats and nonhuman primates, these treatments improve forelimb recovery by approximately 50% compared with lesioned controls (3, 5, 6) but do not support full functional recovery.

Separately from proregenerative strategies, intensive rehabilitation can also improve functional outcomes after CNS trauma (7), driving “plasticity” of *spared* neural circuits that stimulates spared axon sprouting (8–12) and allocation of novel functional roles to existing pathways (13).

To date, it has not been established whether rehabilitation also augments functional outcomes when combined with a proregenerative intervention such as stem cell grafting. Thus, we designed the present experiment to test the hypothesis that forelimb rehabilitation combined with NSC grafting will drive anatomical plasticity of host axons *regenerating* into an NPC graft placed in the lesion site, thereby improving skilled motor grasping performance after midcervical (C5) SCI. Rats underwent a new, more severe cervical injury that substantially compromised bilateral white matter and resulted in persistent 75% loss of skilled grasping; grafting was delayed for 1 full month after injury, a time point at which most humans are admitted to rehabilitation centers after SCI. We found that only the combination of rehabilitation and grafting supports significant recovery of forelimb grasping when both treatments were initiated at a chronic time point of 1 month after injury (*P* < 0.01) and that rehabilitation enhanced regeneration of injured host corticospinal axons into the lesion site (*P* < 0.05).

## Results

### Experimental design.

Four groups of Fischer 344 rats received severe bilateral contusive lesions of the C6 spinal cord: (a) lesion alone (*n* = 20), (b) NPC transplant alone (*n* = 21); (c) rehabilitation alone (*n* = 22); and (d) NPC transplant and rehabilitation (*n* = 22). Four weeks after lesions, all groups underwent reexposure of the lesion, and 2 groups received implants of E14-derived spinal cord NPCs, while the 2 nongrafted groups received injections into the lesion site of the grafting matrix consisting of fibrinogen/thrombin with BDNF, FGF2, VEGF, and MDL28170 (see Methods). Rehabilitation began the next day for 5 days per week; it consisted of forelimb grasping for food reward pellets for 2 hours per day and overnight (14 h/d) exposure to a floor grid containing food reward pellets accessible only by skilled grasping (Supplemental Figures 1 and 2; supplemental material available online with this article; https://doi.org/10.1172/jci.insight.158000DS1). Quantitative assessment of forelimb skilled grasping was performed weekly on the Montoya staircase (Supplemental Figure 3 and ref. 14). Upon completion of behavioral testing, animals underwent anterograde tracing of the corticospinal tract; they were euthanized 2 weeks later.

### Graft survival and fill of the lesion.

**Figure 1:**
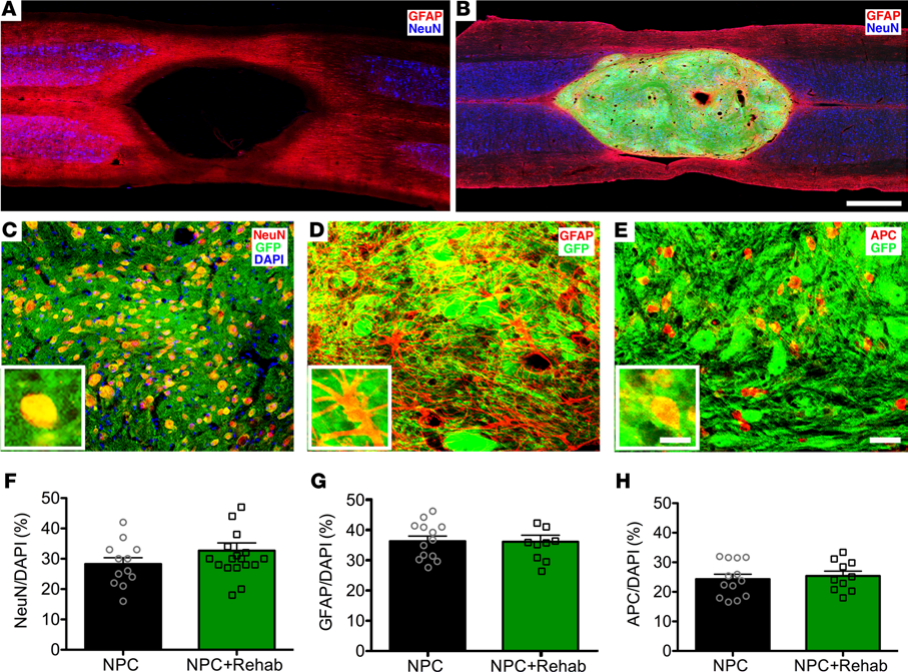
Engraftment and differentiation of transplanted neural progenitor cells. (**A** and **B**) GFAP (red), GFP (green), and NeuN (blue) immunolabeling in the horizontal plane of the C6 bilateral spinal cord contusion. (**A**) Lesion appearance in ungrafted animal near the level of the central canal. There is a large lesion cavity that involves both gray and white matter. (**B**) Transplanted neural progenitor cells (NPCs) (green) survive and fill the large lesion site. (**C**) GFP (green), NeuN (red), and DAPI (blue) triple-fluorescence immunolabeling reveals colocalization of grafted GFP-expressing cells with neuronal marker NeuN. (**D**) GFP (green) and GFAP (red) double-fluorescence immunolabeling reveals colocalization of grafted GFP-expressing cells with GFAP (astrocytes). (**E**) GFP (green) and APC (red) double-fluorescence immunolabeling reveals colocalization of grafted GFP-expressing NPCs with the oligodendrocyte marker APC. (**F–H**) Quantification of differentiation of transplanted NPCs shows that rehabilitation did not significantly alter cell fate reflected in the proportion of graft neurons labeled for (**F**) NeuN, (**G**) GFAP, and (**H**) APC (± SEM; Student’s 1-tailed *t* test). Scale bar: 1 mm (**A** and **B**); 30 μm (**C**–**E**); 10 μm (inset images, **C–E**).

The C6 bilateral contusion resulted in a large lesion cavity that replaced virtually all gray matter and much of the white matter (Figure 1A). The lesion site extended 4–5 mm in rostrocaudal dimension and 2–3 mm mediolaterally. Transplanted NPCs survived and filled the large lesion site in most grafted animals (Figure 1B and Supplemental Figures 4 and 5). In 30 of 43 rats (70%), the transplanted NPCs either completely filled the lesion (24 of 43 rats, 56%; Figure 1B, Supplemental Figures 4 and 5, and Supplemental Table 1) or filled most of the lesion cavity (at least two-thirds filled in 6 of 43 rats, 14%; Supplemental Figures 4 and 5 and Supplemental Table 1). In a minority of animals (13 of 43 animals, 30%), the graft fill of the lesion cavity was less than two-thirds complete, and these animals were excluded from further consideration, because they did not adequately “bridge” the lesion cavity (Supplemental Figures 4 and 5 and Supplemental Table 1).

### Transplanted NPCs adopt neuronal and glial fates and extend large numbers of axons into the host spinal cord.

Transplanted NPCs differentiated into neurons and glia (Figure 1, C–E). 27.9% ± 1.5% of cells in the NPC transplant and rehabilitation group (*n* = 16) and 24.9% ± 1.6% cells in the NPC transplant–alone group (*n* = 14) expressed the neuronal marker NeuN, respectively, a difference that was not statistically significant (*P* = 0.1, 1-tailed Student’s *t* test; Figure 1, C and F). Thus, rehabilitation did not affect cell fate in the graft. Neurons were distributed relatively evenly through the graft (Supplemental Figure 6). The estimated total number of DAPI-labeled grafted cells was 907,000 ± 83,000 in the NPC transplant and rehabilitation group compared with 756,000 ± 78,100 in the NPC transplant–alone group, a difference that was not significant (*P* = 0.1, 1-tailed Student’s *t* test). The estimated total number of neurons per graft was 260,000 ± 22,000 in the NPC transplant and rehabilitation group compared with 213,000 ± 32,000 in the NPC transplant–alone group, a difference that was not significant (*P* = 0.1, 1-tailed Student’s *t* test). 36.4% ± 1.7% of cells in the NPC transplant and rehabilitation group expressed the astrocyte marker GFAP compared with 36.2% ± 2.2% in the NPC transplant–alone group (Figure 1, D and G). The oligodendrocyte marker APC was expressed in 25.4% ± 1.6% of cells in NPC transplant and rehabilitation group and 24.3% ± 1.7% of cells in NPC transplant–alone group (Figure 1, E and H). These percentages account for approximately 90% of all cells in grafts labeled with the nuclear marker DAPI; the remaining 10% of cells in grafts likely consist of endothelial and migrating host cells.

**Figure 2:**
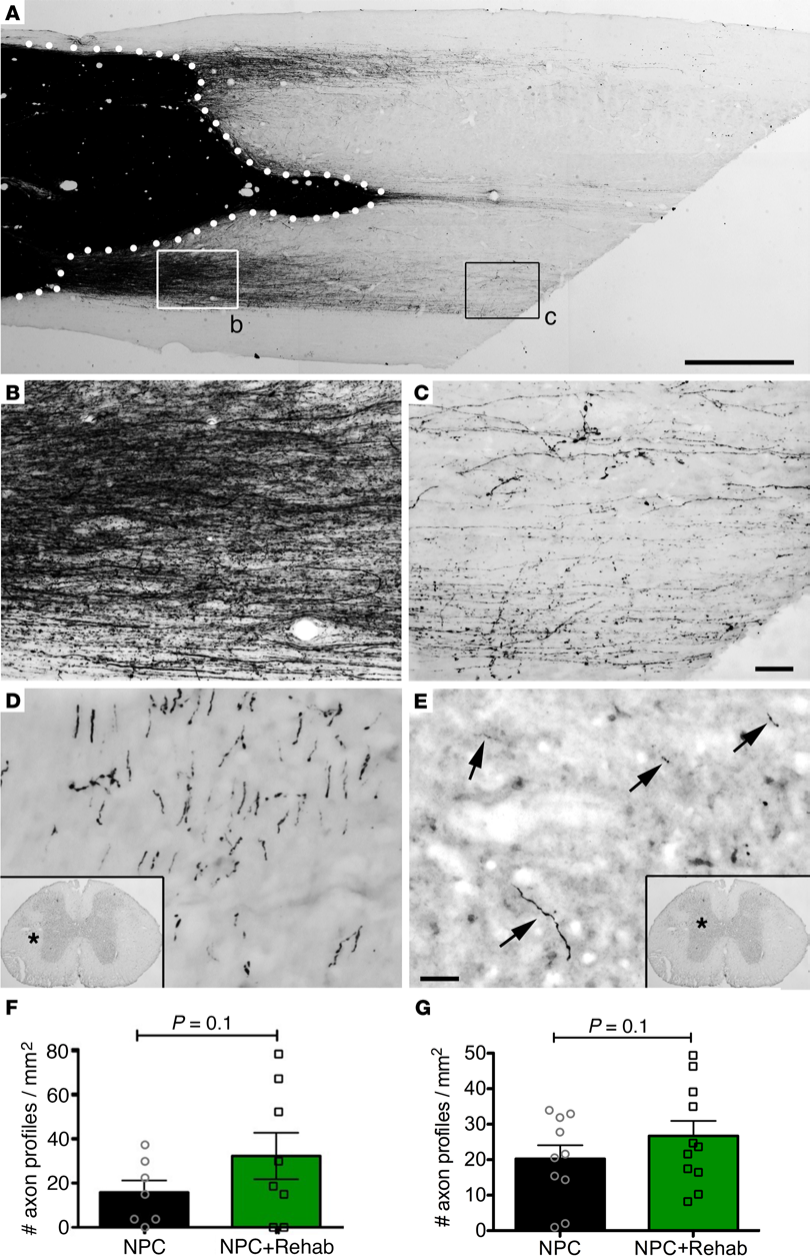
Axonal outgrowth from neural progenitor cell grafts. (**A**) GFP light-level immunolabeling reveals extensive outgrowth of neural progenitor cell (NPC) graft-derived axons into caudal host spinal cord. Dotted lines outline graft within contusive lesion site. Horizontal section; rostral to left. (**B** and **C**) Higher-magnification images of boxed areas in **A** showing (**B**) large numbers of axons near the graft and (**C**) a moderate density of axons more distantly. (**D** and **E**) GFP-labeled axons extend from the C5 lesion site to C8 level (shown) in both (**D**) white matter and (**E**) gray matter. (**F** and **G**) Quantification of axon profiles in host white matter and gray matter at C8. There is a trend toward greater numbers of axons in host white matter 3 spinal cord segments caudal to the graft in animals that underwent rehabilitation (± SEM; Student’s 1-tailed *t* test). Asterisk indicates locations of higher magnification view, and arrows indicate individual axon profiles in host gray matter. NPC graft alone, *n* = 10; NPC and rehabilitation, *n* = 11. Scale bar: 1 mm (**A**); 64 μm (**B** and **C**); 31 μm (**D** and **E**).

Consistent with previous findings (2, 3, 15, 16), GFP labeling demonstrated that grafts extended very large numbers of axons into the host spinal cord caudal to the lesion (Figure 2, A and B), and many of these axons extended over long distances (Figure 2, C–E). Six mm caudal to the lesion within the C8 spinal cord segment that controls distal forelimb grasping, axons were present in most white and gray matter regions (Figure 2, D and E). Quantification of axonal number in mediolateral white matter at C8 revealed a trend toward a doubling in axonal numbers in the NPC transplant and rehabilitation group (32.4 ± 10.5 axons/mm^2^) compared with the NPC transplant–alone group (15.9 ± 5.4/mm^2^) (*P* = 0.1, 1-tailed Student’s *t* test, Figure 2F). There was also a trend favoring increased axonal number in gray matter at C8 in the NPC transplant and rehabilitation group (26.6 ± 4.2 axons/mm^2^) compared with the NPC transplant–alone group (20.2 ± 3.8 axons/mm^2^) (*P* = 0.1, 1-tailed Student’s *t* test, Figure 2, E and G). There was no significant correlation between estimated numbers of neurons in grafts and axon density in host gray matter below the lesion (Supplemental Figure 7).

### Rehabilitation significantly enhances corticospinal axonal regeneration into grafts.

**Figure 3:**
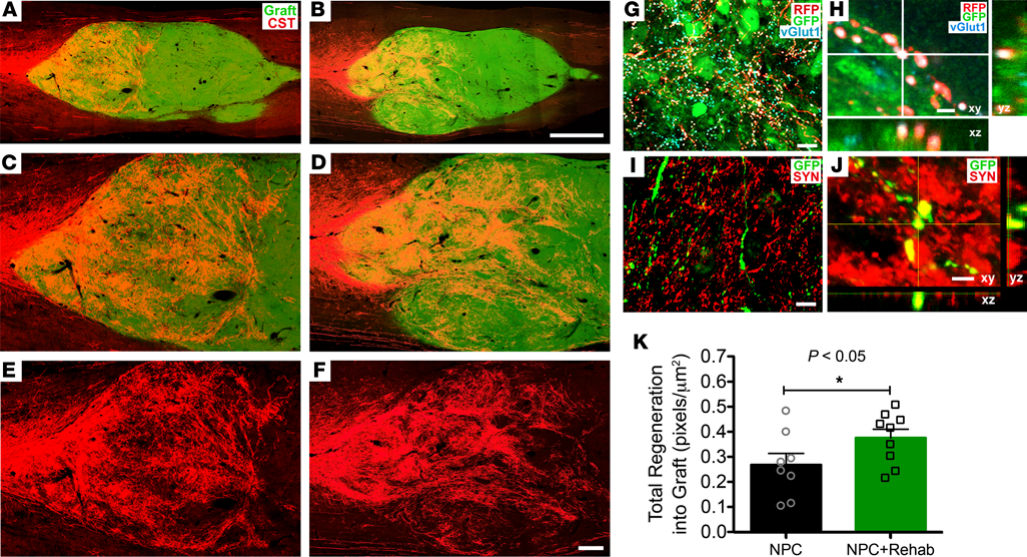
Corticospinal regeneration into neural progenitor cell graft. (**A** and **B**) Corticospinal axons robustly regenerate into the rostral half of a GFP-expressing neural progenitor cell graft (green). Horizontal section; rostral to left and caudal to right. Corticospinal axons were labeled with red fluorescent protein (RFP). (**A**) Neural progenitor cell (NPC) and (**B**) NPC and rehabilitation groups. (**C** and **D**) Higher-magnification images of images in **A** and **B**, and (**E** and **F**) single RFP channel of corticospinal axons. (**G** and **H**) Host corticospinal axons (labeled for RFP) regenerating into grafts form bouton-like structures that contain vesicular glutamate transporter 1 (vGlut1, blue) in close apposition to grafted (GFP-expressing, green) cell processes, suggesting synapse formation. (**I** and **J**) In host gray matter located 3 spinal cord segments below the lesion, GFP-labeled graft axons also form bouton-like structures that colocalize with the presynaptic marker synaptophysin (Syn, red). (**K**) Quantification of total corticospinal axon regeneration into NPC grafts normalized to intensity of corticospinal axon labeling in the dorsal columns at C3. There is significantly greater corticospinal regeneration into NPC graft among animals that underwent rehabilitation (*P* < 0.05, Student’s 1-tailed *t* test). NPC graft *n* = 8; NPC and rehabilitation groups, *n* = 9. Scale bars: 1 mm (**A** and **B**); 250 μm (**C–F**); 30 μm (**G**); 2 μm (**H**); 20 μm (**I**); 4.5 μm (**J**).

Consistent with previous reports, corticospinal axons extensively regenerated into NPC grafts in the lesion site (Figure 3), distributed primarily in the rostral half of the graft (Figure 3, A–F). Quantification of corticospinal axons regenerating into NPC grafts revealed a significant increase in the NPC transplant and rehabilitation group compared with the NPC transplant–alone group (*P* < 0.05, 1-tailed Student’s *t* test; Figure 3K). Corticospinal axons regenerated for distances up to 3 mm into the graft (Figure 3, A–F). Corticospinal axons regenerating into NPC grafts formed bouton-like structures that expressed the presynaptic marker vesicular glutamate transporter 1 (vGlut1) (17); Figure 3, G and H). Moreover, GFP-labeled axons extending from the graft into host spinal cord gray matter at C8 exhibited bouton-like swellings that colocalized with the presynaptic marker synaptophysin (Figure 3, I and J). These findings suggest that there was synaptic connectivity between graft-derived axons and host neurons.

Separately from axonal regeneration, it is possible that rehabilitation could also enhance sprouting of corticospinal axons above the spinal cord lesion (18). However, quantification of corticospinal axon density at C2 in cross sections of the spinal cord showed no differences between the 2 experimental groups of animals that received NPC grafts with or without rehabilitation (Supplemental Figure 8).

### Training and rehabilitation significantly improve functional outcomes.

**Figure 4:**
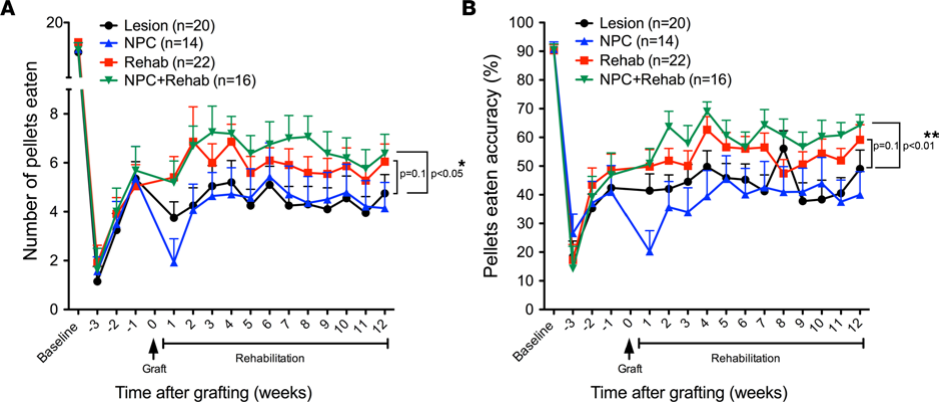
Neural progenitor cells combined with rehabilitation significantly improve functional outcomes. (**A**) Number of pellets eaten on Montoya staircase task. A generalized estimating equation with Poisson distribution (for counting data) and autoregressive correlation matrix structure for repeated measures showed a significant group by time interaction after treatment (Wald χ^2^, 228.2, *P* < 0.01 overall for all groups). Further analysis with generalized estimating equation and post hoc interaction with any 2 groups showed that the neural progenitor cell (NPC) and rehabilitation group differed significantly from the lesion-alone group, with a main effect of condition (Wald χ^2^, 4.54, *P* = 0.03) and a condition by time interaction (Wald χ^2^, 228.2, *P* < 0.001). There was a trend toward better performance, comparing the lesion and rehabilitation group with the lesion-alone group (Wald χ^2^, 4.54, *P* = 0.1); no other group comparisons were significant. Lesion alone, *n* = 20; lesion and rehabilitation, *n* = 22; NPC alone, *n* = 14; and NPC and rehabilitation group, *n* = 16. (**B**) Accuracy of pellet retrieval (number of pellets displaced divided by number of pellets eaten). A generalized estimating equation with γ distribution (for percentage data) and autoregressive correlation matrix structure for repeated measures showed a significant group by time interaction after treatment (Wald χ^2^, 398.2, *P* < 0.01). Further analysis with generalized estimating equation and post hoc interaction with any 2 groups showed that the NPC and rehabilitation group differed significantly from the lesion-alone group (Wald χ^2^, 7.15, *P* < 0.01) and the Lesion-NPC group (Wald χ^2^, 6.43, *P* = 0.01), and showed a trend toward better performance against the lesion and rehabilitation group (Wald χ^2^, 2.77, *P* = 0.1). There was a trend toward better performance comparing the lesion and rehabilitation group with the lesion-alone group (Wald χ^2^, 2.47, *P* = 0.1) as well as lesion and rehabilitation group with the lesion and NPC (Wald χ^2^, 3.32, *P* = 0.07); no other group comparisons were significant.

One week after placement of the C6 bilateral contusion lesion, the ability of rats to grasp and retrieve food pellets was reduced by 90% (Figure 4A), with no differences among groups (ANOVA, *P* = 0.84; Figure 4A). Over the next 2 weeks, all groups improved, yet exhibited a persistent loss of approximately 66%–77% in skilled grasping ability; this was a period in which animals received neither rehabilitation nor grafting. Similar patterns of deficits were observed when quantifying the *accuracy* of food pellet retrieval, i.e., the number of pellets eaten divided by the number of pellets displaced (Figure 4B).

Four weeks after injury, animals received NPC grafts or sham surgery, and rehabilitation was started the next day in 2 groups (NPC transplant and rehabilitation and rehabilitation alone). Over the subsequent 12-week period, significant differences among groups were observed using a Poisson loglinear model with autoregressive correlation matrix structure for repeated measures (*P* < 0.01, group × time interaction after treatment, Wald χ^2^, 228.2; Figure 4A). The NPC transplant and rehabilitation group differed significantly from every other group, with a main effect of group (Wald χ^2^, 4.54; *P* = 0.03) and a group × time interaction (Wald χ^2^, 85.1; *P* < 0.01). No other group exhibited significant and persistent improvements in pellets eaten relative to their own baseline 1 month after lesion (Figure 4A). There was a trend toward better performance in the rehabilitation-alone group compared with the lesion-alone group (Wald χ^2^, 4.54; *P* = 0.1); no other group comparisons were significant.

Similarly, significant differences among groups were observed in pellet retrieval *accuracy* (pellets eaten/pellets displaced) after NPC transplant and rehabilitation using an autoregressive correlation matrix structure for repeated measures (*P* < 0.01, group × time interaction, Wald χ^2^, 398.2). The NPC transplant and rehabilitation group differed significantly from every other group, with a main effect of group (Wald χ^2^, 10.7; *P* < 0.01) and a group × time interaction (Wald χ^2^, 41.3; *P* < 0.005). Accuracy improved by approximately 20% and was maintained through all weeks of testing relative to the pretreatment baseline. No other group exhibited significant and persistent improvements in pellet retrieval accuracy relative to their own baseline after lesion (Figure 4B). There was a trend toward better performance in the rehabilitation-alone group compared with the lesion-alone group (Wald χ^2^, 2.47; *P* = 0.1).

## Discussion

These findings demonstrate for possibly the first time that a proregenerative NPC therapy combined with rehabilitation significantly improved both anatomical and functional outcomes after SCI. While rehabilitation has previously been reported to promote sprouting of spared axons (7, 12, 13, 19), in the present model in which axonal regeneration is possible, rehabilitation enhanced *regeneration*. Moreover, functional outcomes *only* improved among animals receiving both NPC grafts and rehabilitation; either treatment alone was not effective in statistically improving motor outcomes. In the context of these long, 4-week delays before application of treatment and a more severe, bilateral cervical lesion model, *both* NPC grafting and rehabilitation were required to support significant functional improvement.

We note, however, that the functional outcomes of the NPC transplant and rehabilitation and rehabilitation-alone groups did not differ statistically from each other, even though only the NPC transplant and rehabilitation group reached statistical significance in differing from lesioned controls. In fact, the provision of rehabilitation alone starting at a *chronic* time point after injury trended toward improving functional outcomes (*P* = 0.1 compared with lesioned controls), providing evidence of a possible benefit of rehabilitation in chronic SCI. While this study was well powered with *n* = 20 animals per group, further expansion of group sizes would be required to establish whether there is a statistically significant benefit of rehabilitation alone in chronic SCI. The potential benefit of rehabilitation alone merits further study, because a current dogma in the field is that rehabilitation is most likely to be effective when applied in subacute, but not chronic, phases of injury. A possible mechanism underlying rehabilitation-induced improvement in function after chronic injury in the absence of a NSC graft is axonal sprouting, which could occur either at the level of corticospinal sprouting within the spinal cord, or sprouting of corticospinal projections to the red nucleus and the reticular formation. Rehabilitation could also induce functional improvement through sprouting of noncorticospinal systems, including brain stem motor projections (rubrospinal, raphespinal, reticulospinal) and intraspinal systems. In this study, we did not detect sprouting of corticospinal projections to the spinal cord rostral to the lesion, and future studies will accordingly focus on sprouting in other locations.

In previous studies, we have reported beneficial functional effects of NSC grafts alone, but these have generally been after unilateral cervical lesions that are inherently less severe and spare greater amounts of host tissue (3, 5, 6). We have observed functional improvement after grafting NPCs to complete thoracic spinal cord transections on measures of gross locomotion *(*2, 16*)*, but spinal circuitry for hind limb locomotion is contained in lumbar spinal cord segments below a complete transection: simple activation of these circuits is sufficient to enable hind limb movement (20, 21). Neural progenitor grafts placed in thoracic injury sites are capable of propagating gross excitation of host neurons below a lesion (22), and this simple and relatively nonspecific activation may be sufficient to evoke hind limb movement. In contrast, the execution of skilled forelimb grasping is a higher order behavior that requires greater reconstitution of neural circuits supporting behavior. Rehabilitation may represent a means of generating greater reconstitution of neural circuits supporting behavior by stimulating activity after the injury, promoting greater regeneration (shown here) and potentially stabilizing functional useful synapses.

In addition to the fact that cervical lesions generated in this experiment were more severe than in previous experiments, the 1-month delay to treatment is also a longer time gap than studied previously. We recently reported that adult corticospinal neurons regress to an immature transcriptional state after SCI (23); in this state, they are able to regenerate axons into a NPC graft. This window of transcriptional immaturity fades after 2 weeks, likely heralding a subsequent reduction but not elimination of the ability of corticospinal axons to regenerate into stem cell grafts. For this reason, NPCs alone may not have supported functional improvement when grafted 1 month after injury, particularly in the context of a more severe, bilateral contusion model. Indeed, a potential mechanism underlying the benefit of rehabilitation may be to reopen a state of transcriptional immaturity in the corticospinal neuron, enabling greater regeneration and improved functional outcomes.

This is the first study to our knowledge to show a benefit of NPC transplantation, or any experimental treatment, after severe bilateral cervical contusion in the rat model. We chose to use the severe cervical contusion model because it is a clinically relevant model: the majority of human SCI occurs at cervical levels (57%, ref. 24), and most human injuries are severe (82% ASIA A-C; ref. 25). Despite these statistics, the majority of preclinical studies (81%; ref. 26) use thoracic contusion models, and most of these are moderate-force injuries rather than severe. Anderson and colleagues (2009; ref. 27) reported a bilateral cervical contusion injury model in Sprague-Dawley rats that was of moderate severity using impact parameters similar to those of the present study; however, the larger lesion cavities and more severe functional deficits we observed were likely a result of using Fischer 344 rats, which weigh 30% less than Sprague-Dawley rats of the same age. Severe SCI models may be rarely used in the literature because of the difficulty in achieving even partial functional benefits. Yet our study demonstrates the importance of testing in these more severe cervical lesion models and the greater challenges that they pose in attaining significantly improved functional outcomes. We did observe significant behavioral recovery in this more severe lesion model, with an approximate 50% effect size compared with lesioned controls. The magnitude of this effect size is suitable for consideration of translation to human clinical trials.

These findings reflect the importance of focused and intense rehabilitation in combination with a proregenerative therapy in improving outcomes after SCI. The ability of humans to cooperate may extend the effects of rehabilitation beyond those observed in this study, because patients can follow instructions to maximize the amount and focus of rehabilitation after NSC grafting.

### Conclusions.

The combination of proregenerative therapies and rehabilitation results in significantly greater anatomical and functional recovery after SCI compared with either alone. These results indicate the importance of incorporating routine, intensive, and coordinated rehabilitation strategies into the clinical design of candidate therapies for CNS disorders that advance to human trials.

## Methods

### Animals.

One hundred and fifty adult female Fischer 344 rats (Envigo), weighing 150–200 g, were used for this study. Animals had free to access to food and water throughout the study; among animals undergoing rehabilitation, food was freely available through the training tasks and animals were weighed weekly to ensure they did not lose weight. For all surgeries and perfusion animals were deeply anesthetized using a combination (2 mL/kg) of ketamine (25 mg/mL), xylazine (1.3 g/mL), and acepromazine (0.25 mg/mL).

### Behavioral training.

Rats underwent training on 3 behavioral tasks prior to placement of spinal cord lesions. The Montoya staircase was used to assess forelimb reaching performance (Montoya, 1991; ref. 14; Lafayette Instrument Co.). Rats were habituated to the apparatus for 1 week and were then trained to grasp 2 food pellets (BioServ-F0042; 45 mg sucrose) placed onto each of 7 steps. Training comprised two 15-minute sessions daily (total 30 min/d) for 15 days. The maximum number of food pellets eaten was 22 using both forelimbs. Of 150 rats, 102 rats (68%) were able to successfully attain a minimum criterion performance level of 8 or more food pellets retrieved from the staircase device to advance in this study. For assessment, the food-deprived rats were given 15 minutes to retrieve and eat as many sugar pellets as possible from the 2 sides of each step. The number of pellets eaten and displaced was recorded. “Accuracy” was calculated as the number of food pellets eaten divided by the number of food pellets displaced and expressed as a percentage.

Rats also had 5-day-per-week exposure to a “rehabilitation cage,” consisting of a cage with wide slits (Supplemental Figure 1A) through which the rat could reach to grasp and retrieve food pellets (BioServ, Dustless Precision Pellets, 45 mg, regular diet, 4 mm in diameter). Food pellets were placed on a tray located 1 cm away from the side of the cage, out of range of the tongue (Supplemental Figure 1B). To successfully retrieve the pellet, the rat had to grasp the pellet, carry it over the gap, and eat it. This is modified from a device designed by Whishaw and colleagues (Whishaw, 2000; ref. 28). The food tray was 17 cm high × 6.5 cm wide × 1.2 cm high, and the cage was 51 cm long × 31 cm wide × 33.5 cm high (Supplemental Figure 1A). Rats were exposed to this cage for 5 days per week, 2 hours per day. Twelve rats were run simultaneously in side-by-side cages, and the number of food pellets retrieved and eaten were not systematically quantified. However, to gauge each rat’s engagement with the protocol, we weighed each food tray at the end of the session to determine the weight of pellets displaced from the tray. These findings are presented in Supplemental Figure 1C.

To provide additional rehabilitation for 5 days per week, rats had overnight exposure to a metal grid box based on a design by Fawcett and colleagues (7) (Supplemental Figure 1, D and E). The grid box contained the same food pellets as the rehabilitation cage. The grid consisted of 1.2 × 1.2 cm square openings and was 2.5 cm deep, and rats could not retrieve the food pellets with their tongue. It was filled with 50 g of food pellets each evening and was weighed at the start and conclusion of the overnight session (Supplemental Figure 1F). The 21 cm long × 15 cm wide box fit into the home cage and provided an opportunity for grasping during the normal nocturnal period of rat activity. Rats that received rehabilitation were housed individually to avoid competition for food pellets.

Preoperative training was performed over 2 weeks prior to placement of spinal cord contusions.

Rats began rehabilitation 4 weeks after spinal cord lesions and 1 day after grafting or sham surgery. Postlesion rehabilitation consisted of the same schedule as above: rehabilitation cage for 2 hours per day and overnight grid box for 5 days per week. Nonrehabilitated animals were kept in the home cages with free access by mouth to Envigo Teklad Laboratory Diet food pellets placed on the top of the cage lid with stainless steel wire bars.

### Bilateral C6 spinal cord contusions.

A dorsal laminectomy was performed to expose the C6 spinal cord. A bilateral contusion was administered using an Infinite Horizons Impactor (Precision Systems & Instrumentation) set to 200-kilodyne force with 1-second dwell time and an impactor head of 3.5 mm diameter (total *n* = 150; Supplemental Table 1). Animals received injections of lactated ringers solution (30–50 mL/kg), banamine (2.5–5 mg/kg), ampicillin (3–5 mg/kg/), and buprenorphine (0.025 mg/Kg) around the clock for 3 days. Nutri-cal (2 mL, Henry Schein) and DietGel Recovery (56 g, ClearH_2_O) were administered daily for 7 days. 148 rats survived the first postoperative week. Postlesion forelimb reaching was measured 1, 2, and 3 weeks after lesion. After lesion, at week 3 animals were pseudorandomly divided into 1 of 4 groups matched for food pellet grasping performance on the Montoya staircase, ensuring distribution into 4 groups with similar degrees of deficit (Figure 4A): (a) lesion alone (*n* = 20), (b) NPC transplant alone (*n* = 21); (c) rehabilitation alone (*n* = 22); and (d) NPC transplant and rehabilitation (*n* = 22).

Four weeks after lesions, primary E14 rat spinal cord NPCs were grafted into the lesion site in the NPC transplant–alone and rehabilitation NPC transplant and rehabilitation groups. Cells were grafted in a fibrinogen/thrombin solution containing BDNF (50 μg/mL), FGF2 (10 μg/mL), VEGF (10 μg/mL), and an antiapoptosis small molecule, MDL28170 (50 μM). Previous work determined that this grafting cocktail is necessary to support graft survival in larger lesion cavities (15). Cells were grafted at a concentration of 250,000 cells/μL in a total volume of 7 μL through the dura and into the lesion cavity using pulled glass micropipettes of outer diameter 100 μm. Seven injections were made over the rostral-to-caudal length of the lesion 0.7 mm lateral to midline bilaterally, and 1 addition injection was placed into the center of the lesion. The 2 nongrafted groups received injections of the grafting cocktail without cells.

Two weeks prior to sacrifice, all animals underwent anterograde tracing of the corticospinal tract using intracortical injections of AAV8 viral vectors expressing membrane-targeted TdTomato (29). Vectors were injected bilaterally into forelimb and hind limb areas of primary motor cortex and primary somatosensory cortex at the following coordinates relative to bregma: A/P + 1.2 to −2.8 mm; M/L ± 1.7 to 3.7 mm; and depth 1.2 to 1.5 mm.

### Preparation of E14-derived multipotent NPC grafts.

As previously described (2), spinal cords from E14 transgenic Fischer 344 rat embryos constitutively expressing GFP under the ubiquitin C promoter (30, 31) were dissected and dissociated. GFP-expressing donor cells were used to readily detect grafted cells placed into wild-type hosts that did not express GFP. Dissected cords were placed in a 15 mL conical tube containing 1 mL of ice-cold Hank’s Balanced Salt Solution. The embryonic spinal cord was digested chemically with 0.125% trypsin at 37°C for 12 minutes following by mechanical dissociation into single-cell suspension (32). E14 spinal cords at this stage mainly consist of lineage-restricted NPCs but also small numbers of NSCs and young neurons (33). Approximately 1.5 millions of cells were grafted into the lesion site mixed with growth factor at a total volume of 7 μL. We chose the term NPCs to represent dissociated E14 early stage neural cells.

### Histology and immunolabeling.

Animals were transcardially perfused with 4% paraformaldehyde. Blocks of cervical spinal cord 1.5 cm long centered around the C6 lesion were sectioned in the horizontal plane on a cryostat set to 30 μm thickness. In addition, C2 and C8 spinal cord levels were sectioned in the coronal plane on a cryostat set to 30 μm thickness. Sections were labeled for (a) GFP rabbit polyclonal antibody (A6455; Invitrogen, Thermo Fisher Scientific; 1:3,000) or GFP chicken polyclonal antibody (ab13970; Abcam; 1:3,000) to assess graft survival, differentiation, and process outgrowth; (b) RFP rabbit polyclonal antibody (Rockland, 600-401-379, 1:5000) to amplify TdTomato signal for corticospinal tract tracing; (c) NeuN guinea pig polyclonal antibody (ABN90; MilliporeSigma; 1:1,000) to label mature neuronal nuclei; (d) GFAP mouse monoclonal antibody (MAB360, clone GA5, EMD Millipore; 1:1,500) to label astrocytes; (e) APC mouse monoclonal antibody (OP80, clone CC-1; Oncogene; 1:800) to label oligodendrocytes; (f) mouse monoclonal synaptophysin antibody (MAB5258, clone SY38; EMD Millipore; 1:1,000) to label presynaptic proteins; and (g) rabbit polyclonal vGlut1 antibody (V0389-200, MilliporeSigma, 1:1,000). After incubation in primary antibodies, sections were incubated with Alexa 488–, 594–, or 647–conjugated donkey secondary antibodies (Invitrogen, Thermo Fisher Scientific; 1:500) and were coverslipped with Fluoromount-G (SouthernBiotech).

### Image analysis.

For examination and quantification of GFP-labeled axons, light-level GFP immunolabeling was performed in every sixth section incubated overnight at 4°C with GFP primary antibody (rabbit at 1:3,000) and then with HRP-conjugated secondary antibodies (1:50, Vector Laboratory) for 1 hour at room temperature. Diaminobenzidine (0.05%) and nickel chloride (0.04%) were used as chromogens, with reactions sustained for 10 minutes at room temperature. The number of differentiated neurons labeled with NeuN, astrocytes labeled with GFAP, and oligodendrocytes labeled with APC within each NPC graft was quantified in a box of fixed size (1,600 × 1,200 pixels) at ×200 magnification within the graft. This number was divided by the total number of cells per sample box labeled with DAPI. To estimate total number of cells per graft, we divided the total number of DAPI-stained cells in the sampled box by the sampling fraction (the percentage of total graft area that the sample box represented) and multiplied this number by the section sampling frequency (1-in-36 sections). The total number of neurons per graft was estimated by multiplying total number of cells per graft by the proportion of neurons among total DAPI-labeled cells. All quantification was conducted by an examiner, with group identity blinded (2).

The number of GFP-labeled axons extending caudally from NPC grafts to the C8 spinal cord level was quantified in both white matter and gray matter in 1 coronal section per animal. The number of GFP^+^ axons within a 1 square millimeter (mm^2^) area in the intermediate bilateral white matter was quantified, together with all graft-derived axonal profiles in the entire gray matter. Mean values were calculated per group ± SEM.

For quantification of regenerated corticospinal axons into the NPC graft, a series of 5 to 8 horizontal spinal cord sections spanning the dorsal-ventral interface and 1 cross-sectional spinal cord at C2 level were imaged at ×10 magnification and autostitched using a Zeiss Axion Scan Z1 microscope and the software module Zen slidescan.

For each animal, the entire GFP-expressing graft area was sampled and the percentage of the graft occupied by corticospinal axons was measured using ImageJ software (version 1.38D; NIH). To normalize values for efficiency of corticospinal tracing per animal, the total percentage of CST-labeled axons in grafts was divided by the number of corticospinal axons in the main tract at C2.

To quantify corticospinal axon sprouting rostral to the injury, coronal sections at C2 labeled for RFP were imaged at C2 coronal at ×100. Using ImageJ, the entire gray matter region was outlined, and thresholding values on images were chosen such that only RFP-labeled corticospinal axons were measured, and light nonspecific background labeling was not detected. To normalize values for efficiency of corticospinal tracing, the total labeled CST pixels in each animal were divided by the number of corticospinal axons in the main tract of the same section.

### Statistics.

In all quantification procedures, observers were blind to the nature of the experimental manipulation. Analyses were performed by in-house statisticians using IBM SPSS (v.27). For behavioral studies, generalized estimating equations were used to assess group differences in the number and accuracy of pellets eaten after treatment. This is a robust approach similar to mixed repeated measures 2-way ANOVA but applicable to data that are nonnormal. A Poisson loglinear model (for count outcomes) with autoregressive correlation matrix structure for repeated measures was used for the number of food pellets eaten. A γ distribution with loglink function (for percentage outcomes) with autoregressive correlation matrix structure for repeated measures was used for the accuracy of food pellets eaten. Comparisons between 2 groups were tested by 1-tailed Student’s *t* test (JMP software), at a designated significance level of *P* < 0.05. Data are presented as mean ± SEM. JMP Bivaiate fit analysis was used to estimate the correlation between number of neurons in the graft and the number of graft-derived axon profiles at C8 spinal cord gray matter.

### Study approval.

All experiments were conducted in strict accordance with NIH laboratory animal care and safety guidelines. All animal procedures were approved by the Institutional Animal Care and Use Committee of the Department of Veterans Affairs San Diego Healthcare System (San Diego, California, USA).

## Author contributions

PL, CMDF, and MHT designed the research studies, reviewed the data analysis, and wrote the manuscript. PL, CMDF, LG, ANT, AV, and DY conducted the experiments and acquired the data. JRH and ARF did the statistical analysis.

## Supplementary Material

Supplemental data

Supplemental video 1

## Version 1 08/22/2022

Electronic publication

## Version 2 02/03/2023

video added to supplemental data

## Version 3 02/13/2023

Revised correct video to replace existing
