# Supplementary material for: Rehabilitation combined with neural progenitor cell grafts enables functional recovery in chronic spinal cord injury
Source: JCI Insight. 2022 Aug 22;7(16):e158000. doi: 10.1172/jci.insight.158000 (PMC9462483; doi:10.1172/jci.insight.158000)
Supplement: Supplemental data [file jciinsight-7-158000-s005.pdf]

Supplementary figures

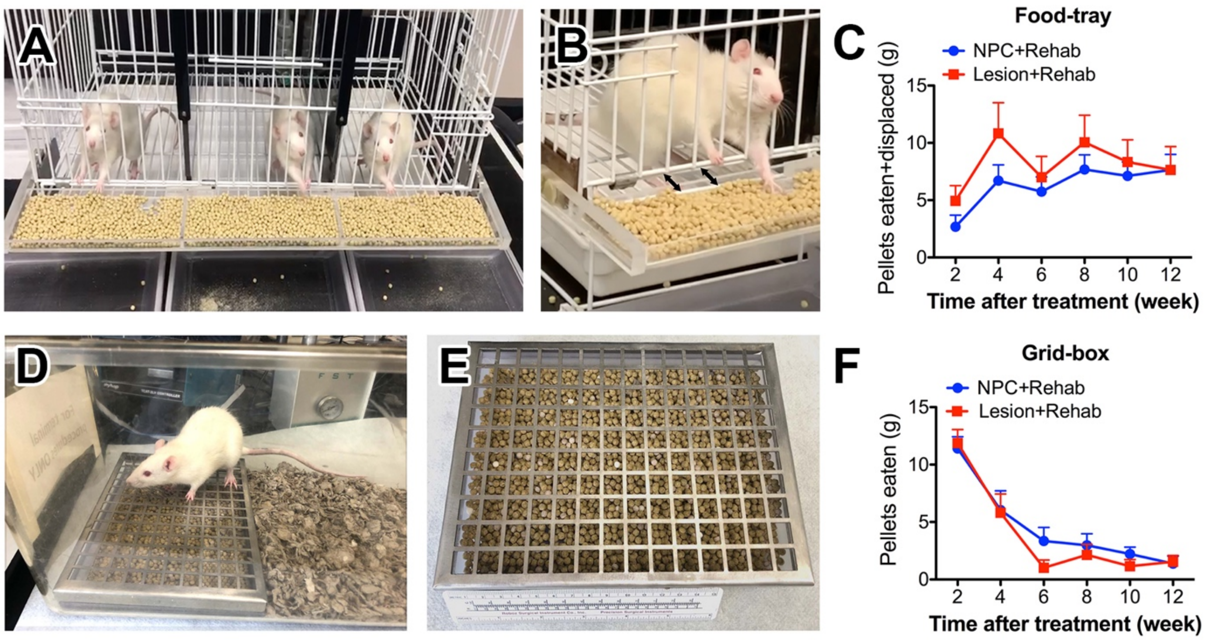

Suppl. Fig. 1. Rehabilitation Training Apparatus and Amount of Food Consumed
(**A)** A high throughput pellet retrieval task allows rats to train for two hours per day ("Food-Tray Task"). A food tray is placed in front of each rat, separated from its neighbors. To retrieve the food reward, the rat must grasp the pellet. (**B**) A 1cm gap between the tray and the cage is present (indicated by black double headed arrow), thereby requiring rats to grasp and retrieve the pellet in order to consume it rather than drop the pellet into the tray below. Measuring the weight of each tray holding the food pellets at the start and the end of the session indicates the number of pellets retrieved. Measuring the weight of the tray underneath the animal at the start and end of the session indicates the number of pellets displaced but not consumed. (**C**) Total of all pellets both retrieved and displaced for the NPC + Rehab vs. the Rehab-Only group. There is a trend toward a greater number of total pellets in the NPC + Rehab group, consistent with findings on the more detailed Montoya staircase ($P<0.05$). The Food-Tray task is a training task; the formal determination of efficacy between treatment groups is based on performance on the Montoya staircase (**Fig 4**). Rats received two hours of exposure to this task for 5 days per week. Modified from Whishaw et al, 1999. (**D-E) I**n addition to the Food-Tray Task, rats had the opportunity to retrieve food pellets from the floors of their cages overnight in the "Grid Box Task". This box measures 21 x 15 cm with 1.2 x 1.2 cm square openings, and is 2.5 cm deep. The grid-like apparatus was loaded with chow pellets and placed on the bottom of the cage each day at 5 PM, and was retrieved and weighed the next day at 9 AM. This was done 5 days per week. This task provided an additional opportunity for rats to engage in forelimb rehabilitation during their normal wake (nocturnal) cycle. Rats were housed one per cage. **(F)** Total pellets removed from the box in grams: the two rehabilitated groups did not differ significantly ($P<0.05$, Student's t-test).

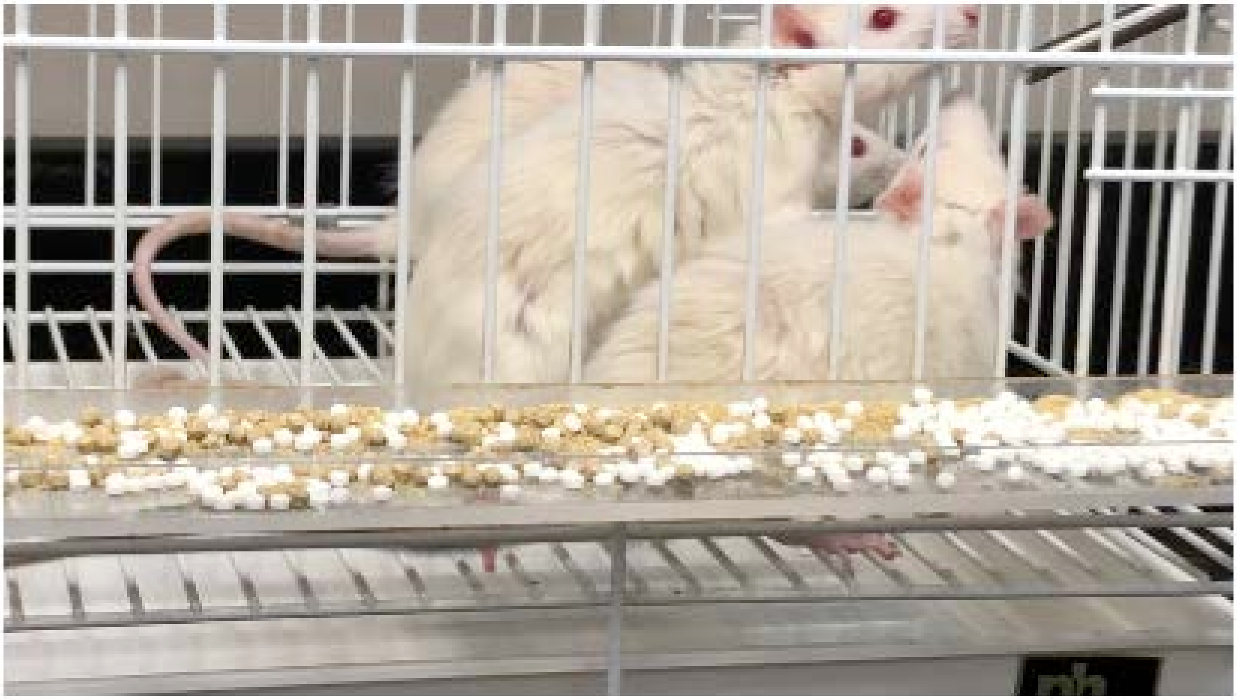

Suppl. Fig. 2. Rehabilitative Training Rehab. Cage. Image from Video Recording of Food Grasping from the Food Tray Task. See related video at: https://insight.jci.org/articles/view/158000/sd/2

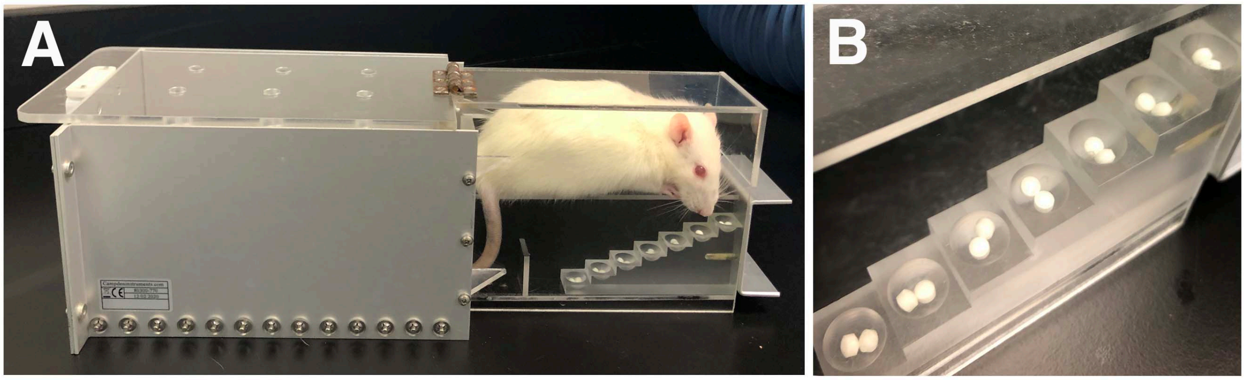

Suppl. Fig. 3. Functional Outcome Assessment on the Montoya Staircase

(A) The staircase apparatus (Model 80301, Lafayette Instrument Company) was used to assess rat skilled forepaw pellet retrieval. Rats can grasp and retrieve pellets placed into each step of the staircase on two sides (2 pellets/step, total 14 pellets/each side). **(B)** Staircase steps.

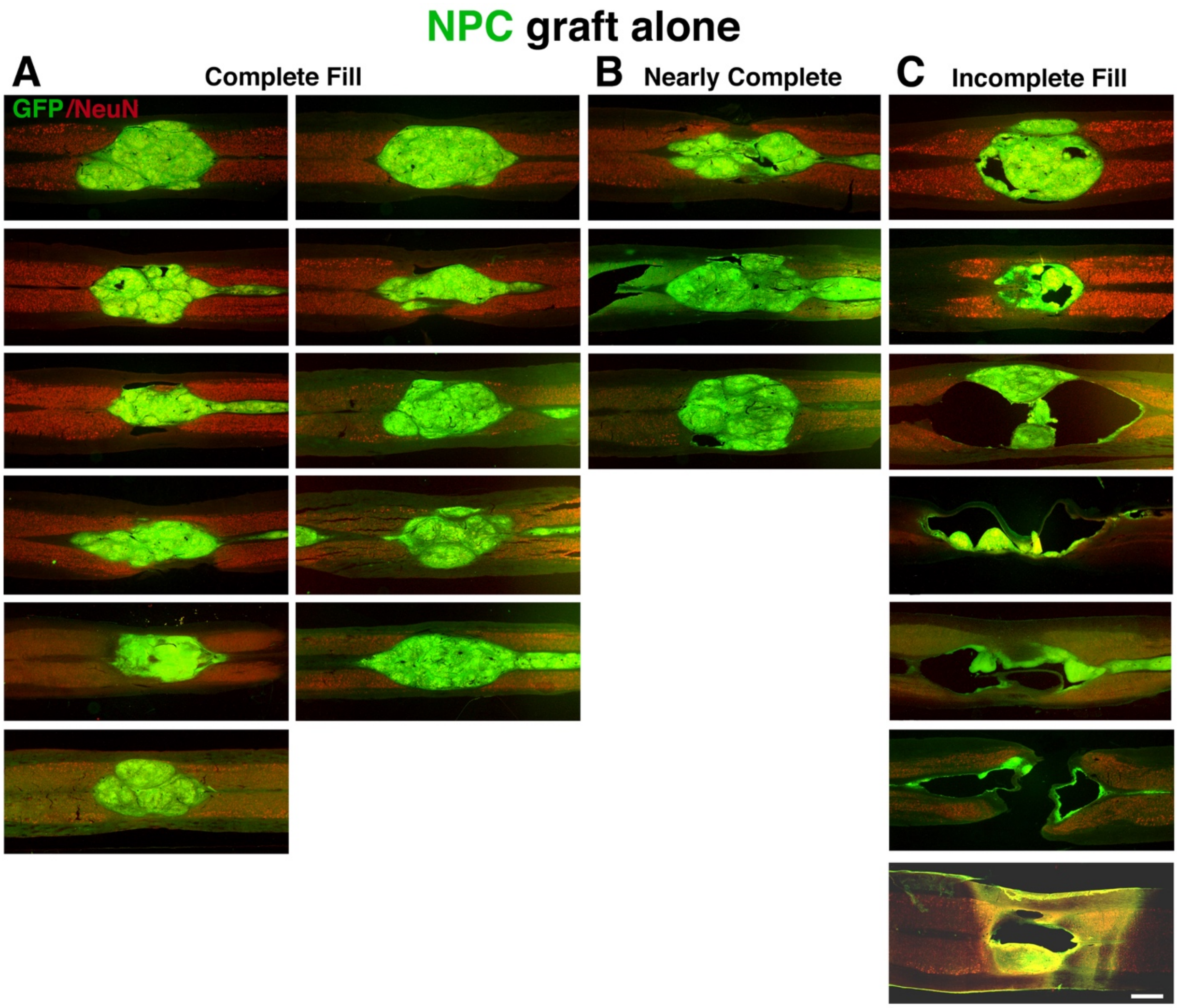

Suppl Fig. 4. Neural Progenitor Graft Survival and Fill of the Contusive Injury Site in Every Animal in NPC Graft-Only group
(A). GFP and **NeuN** double immunolabeling show graft survival and fill of the lesion site by **GFP**-expressing NPC grafts (N = 11). **(B)** Examples of NPC graft survival and fill described as “Nearly Complete” (Table 1). These animals were included in final behavioral and anatomical analyses (N = 3). **(C)** Examples of NPC grafts with incomplete fills of the lesion cavity that were excluded from further analyses (N = 7). Scale bar = 1 mm.

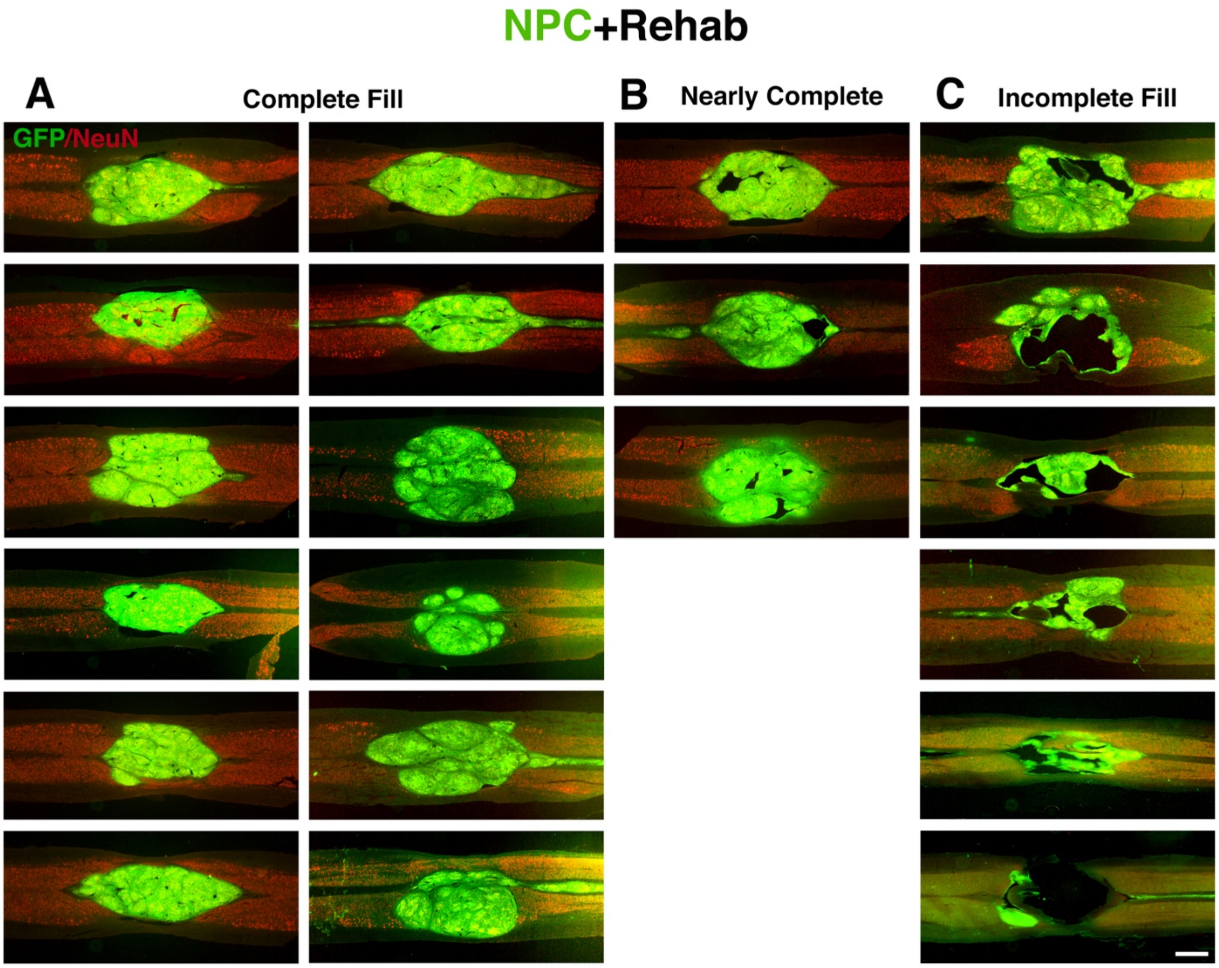

Suppl Fig. 5. NPC graft survival and filling of contusive injury site in every animal in NPC Graft + Rehabilitation group
(A). GFP and **NeuN** double immunolabeling show graft survival and fill of the lesion site by **GFP**-expressing NPC grafts (N = 12); one sample was taken from electron microscopy). **(B)** Examples of NPC graft survival and fill described as “Nearly Complete” (Table 1). These animals were included in final behavioral and anatomical analyses (N = 3). **(C)** Examples of NPC grafts with incomplete fills of the lesion cavity that were excluded from further analyses (N = 6). Scale bar = 1 mm.

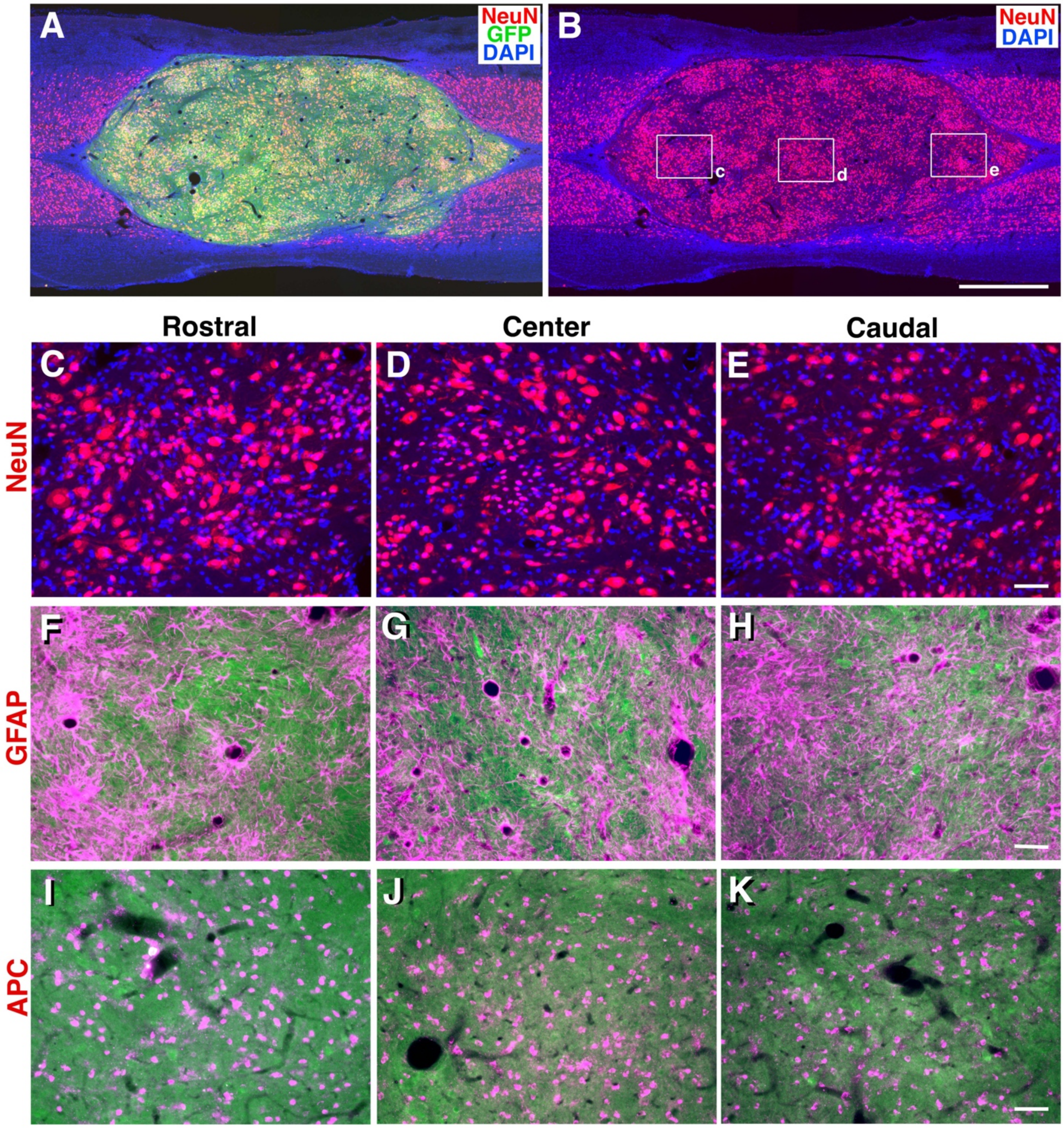

Suppl Fig. 6. Distribution of neurons, astrocytes and oligodendrocytes in NPC grafts
(A-B). GFP, NeuN and DAPI immunolabeling show distribution of NeuN+ neurons in GFP-expressing NPC graft. **(C-E)** Higher magnification images from the boxed area represented rostral, center, and caudal regions of the graft from panel B. **(F-H)** GFAP and GFP labeling show distribution of GFAP+ astrocytes from (**F**) rostral, (**G**) center, and (**H**) caudal region of the GFP-expressing graft. **(I-K)** APC and GFP labeling show distribution of GFAP+ astrocytes from (**I**) rostral, (**J**) center, and (**K**) caudal region of the GFP-expressing graft. Scale bar = 1 mm (A-B); 60 µm (C-K).

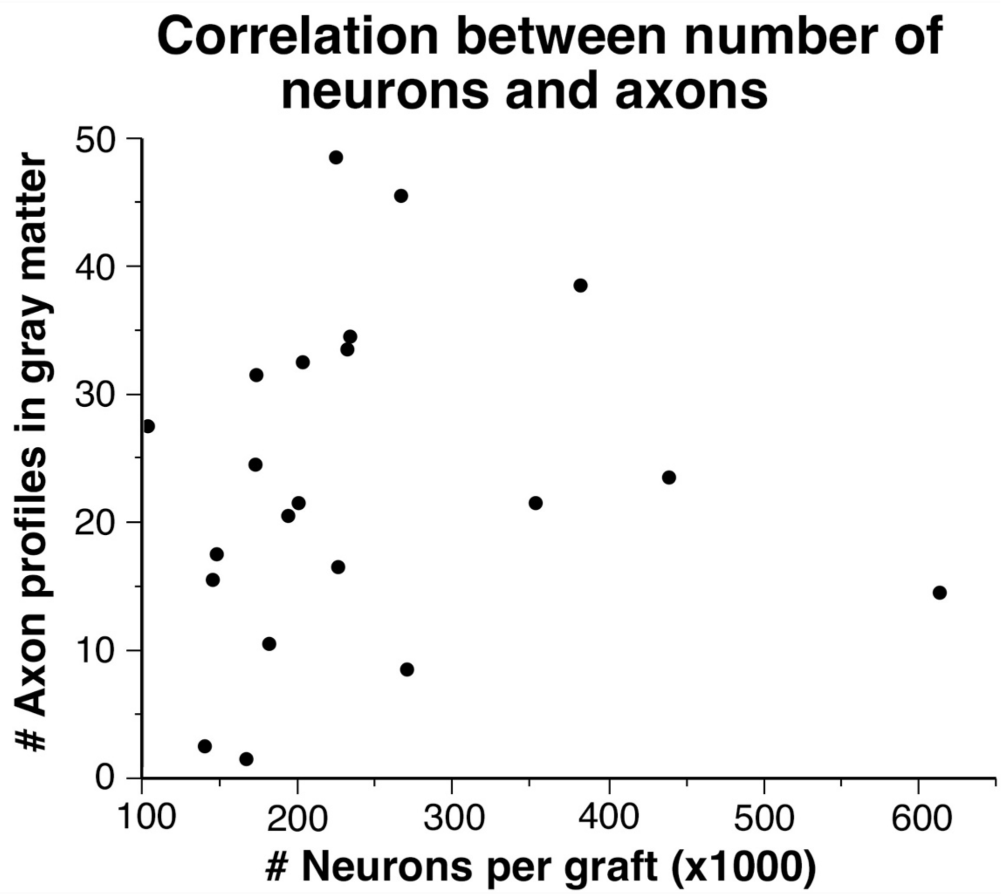

Suppl Fig. 7. Correlation between the estimated number of NeuN positive neurons per graft and the axon profile number in the host gray matter at C8 level. There is no significant correlation (Correlation = 0.09; Significance probability = 0.69, JMP Bivariate Fit analysis).

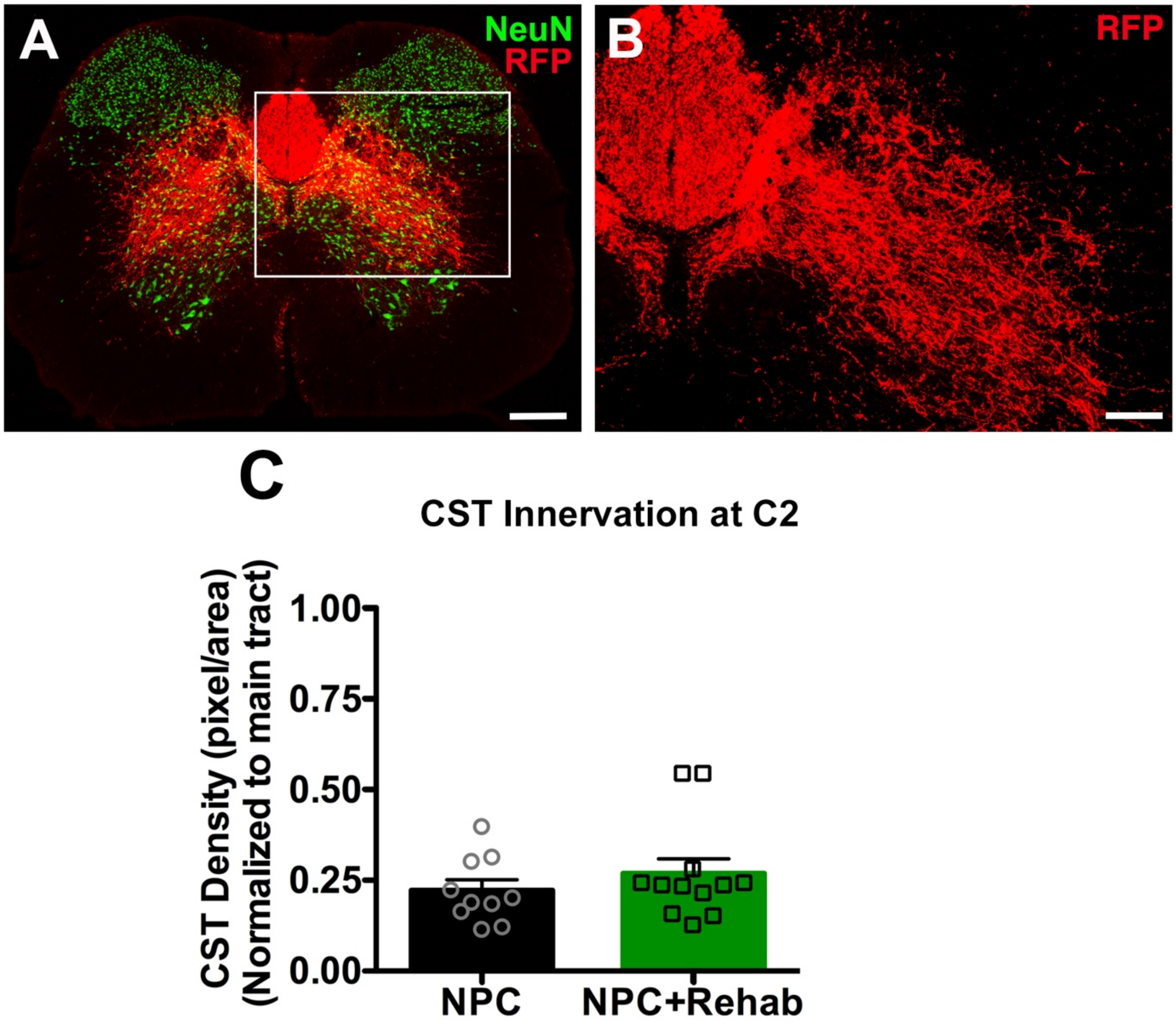

Suppl Fig. 8. Assessment of Corticospinal Axon Sprouting Above the Injury
To determine whether rehabilitation exerted a detectable effect on the sprouting of corticospinal axons rostral to the level of the injury, we quantified the density of corticospinal axons in host gray matter at the C2 level, 3 spinal cord segments above the injury. Corticospinal-labeled pixels per area were quantified. (**A**) Double labeling of **RFP**-labeled **(red)** corticospinal axons at C2 in a coronal section and **NeuN (green)** labels host gray matter region. **(B)** Higher magnification view from box in (**A**) showing **RFP-l**abeled corticospinal collaterals. These regions were compared in all grafted animals. **(C)** Quantification in NPC graft alone (N=10) and NPC + Rehab groups (N=12) (P = 0.09, one-tailed t-test). Scale bars: A=300 µm and B=120 µm.

Table 1, Rehabilitation Experimental Groups

Staircase trained	Learned	Survived C6 Contusion	Groups Assigned		
			**Group**	**Subject #**	**Graft Quality**
N = 150	N = 102 (68%)	N = 85 (83%)	NPC + Rehab	N = 22	Acceptable (16)*<br>Complete fill (13)<br>*Nearly complete fill (3)<br>Incomplete fill (6)
			NPC alone	N = 21	Acceptable (14)*<br>Complete fill (11)<br>*Nearly complete fill (3)<br>Incomplete fill (7)
			Rehab alone	N = 22	N/A
			Lesion alone	N = 20	N/A

*Nearly complete fill indicates that at least two-thirds of the lesion site contained continuous graft spanning the rostral-to-caudal length of the lesion cavity (Fig 1). Animals with complete or nearly complete fill were included in final analyses. A total of 13 subjects were excluded (6 in the NPC+Rehab group and 7 in the NPC group).
